# The Effect of Titanium Dioxide Nanotubes and Graphene Compounds on the Proliferation and Osteogenic Differentiation of Rat BMSCs

**DOI:** 10.3390/jfb16110413

**Published:** 2025-11-05

**Authors:** Chenyuan Zhu, Yuwei Deng, Jing Xu, Jin Wen, Qingfeng Huang, Weiqiang Yu

**Affiliations:** 1 Department of Prosthodontics, Shanghai Ninth People’s Hospital, Shanghai Jiao Tong University School of Medicine, College of Stomatology, Shanghai Jiao Tong University, Shanghai 200011, China; 2 National Center for Stomatology, National Clinical Research Center for Oral Diseases, Shanghai Key Laboratory of Stomatology, Shanghai Research Institute of Stomatology, Shanghai 200011, China

**Keywords:** osseointegration, surface modification, graphene, nanotubes

## Abstract

Graphene-based nanomaterials, including graphene oxide (GO) and graphene quantum dots (GQDs), exhibit exceptional properties, which might facilitate the functional modification of TiO_2_ nanotubes (NTs) for enhanced rapid osseointegration. This study investigated the effects of GO/GQD-deposited TiO_2_-NTs on cell proliferation, osteogenic differentiation of rat bone marrow-derived mesenchymal stem cells (BMSCs), and early osseointegration in male 6-week-old Sprague Dawley (SD) rats. TiO_2_-NTs (control group) were fabricated on titanium substrates via anodic oxidation. GO and GQDs were electrochemically deposited onto the TiO_2_-NTs using cyclic voltammetry with 0.5 mg/mL GO and 0.1 mg/mL GQD dispersions to form NT-GO and NT-GQDs. In vitro assays evaluated cell adhesion, proliferation, and osteogenic differentiation. Implants were randomly inserted into one femoral epiphysis of nine rats (*n* = 3), and osseointegration was evaluated using micro-computed tomography and sequential fluorescence labeling at 2, 4, and 6 weeks post-implantation. Statistical analysis was conducted using ANOVA. Cyclic voltammetry successfully synthesized NT-GO and NT-GQDs, with Raman spectra confirming D and G bands. Both NT-GO and NT-GQDs exhibited superior cell adhesion, proliferation, and enhanced osteogenic differentiation compared with TiO_2_-NTs. Notably, the NT-GQDs significantly promoted new bone formation in vivo. The integration of graphene nanomaterials onto TiO_2_-NTs improves biocompatibility and accelerates osteogenesis, suggesting a promising strategy for enhancing osseointegration in orthopedic and dental implants.

## 1. Introduction

With the global rise in the aging population and the high prevalence of orthopedic conditions such as fractures, osteoporosis, and tooth loss among the elderly, the clinical demand for orthopedic implants or prostheses has grown substantially. Biomedical materials, particularly titanium alloys, are increasingly utilized in orthopedic surgeries and dental implantation procedures [1]. Surface modification of titanium implants plays a critical role in promoting osseointegration, enhancing implant stability, and ensuring long-term clinical success. Early implant failure remains common, especially in patients with systemic conditions such as uncontrolled diabetes, osteonecrosis of the jaw, or bone malignancies [1,2,3]. Medications like proton-pump inhibitors and selective serotonin reuptake inhibitors (SSRIs) may also impair implant osseointegration, the process by which an implant directly integrates with surrounding bone tissue [1,2]. Significant challenges remain in the surface modification of titanium materials.

Among various surface modification strategies, nanomodification has emerged as a highly effective approach for enhancing osseointegration, providing highly effective coating solutions for titanium-based dental implants through significant improvements in their bone integration capabilities [3,4,5]. Early studies have demonstrated that titanium dioxide (TiO_2_) nanotube arrays exhibit excellent osteogenic properties, owing to their ordered structure, high surface area, surface roughness, and capacity as drug delivery carriers. These nanostructures have been shown to possess favorable biocompatibility and to promote the osteogenic differentiation of bone marrow stromal stem cells (BMSCs) [2,3]. Furthermore, nanomaterials can be integrated with reinforcing auxiliary compounds to form multifunctional platforms capable of inhibiting bacterial infection, promoting osteogenesis, and enhancing antitumor activity [6,7,8].

Graphene and its derivatives (GDs) have been widely studied for drug delivery, cancer treatment, biosensing, and tissue engineering in vitro and in vivo [9,10], making them promising for modifying TiO_2_ nanotubes (NTs) to accelerate rapid osseointegration. Among GDs, graphene oxide (GO) and graphene quantum dots (GQDs) have attracted significant attention due to their outstanding properties. GO exhibits good biocompatibility, a large specific surface area, and abundant oxygen-containing functional groups that can serve as active sites for further functionalization [11,12]. GQDs are derivatives of GO and possess quantum size and edge effects, retaining GO’s advantages while offering enhanced solubility, low cytotoxicity, and excellent biocompatibility [13,14]. When combined with metals, polymers, or minerals, GDs often show improved mechanical properties and bioactivity [6,10,15] and potential to promote the adhesion, proliferation, and differentiation of osteoblasts and mesenchymal stem cells (MSCs) [12]; accelerate bone regeneration and repair in bone defects [9,16]; exhibit antibacterial properties [11,17]; and provide immunomodulation [18].

GO and GQDs are studied mainly in solution or single-layer graphene films, but there are limited studies on the modification of metal surfaces, especially titanium surfaces. Xu D et al. reported that sandblasted, large-grit, and acid-etched (SLA)-treated Ti6Al4V, after immersion in GQD solution at a concentration of 0.1 μg/mL, exhibited accelerated new bone formation [14]. Guo J et al. synthesized graphene oxide (GO) on cobalt–chromium–molybdenum alloy via electrodeposition, offering a promising solution to poor bone integration in dental implants [19]. Li et al. reported that graphene, when applied as a 100% continuous film on the titanium surface, enhances cell adhesion, proliferation, and osteogenic differentiation [20]. Liu Y et al. employed chemical vapor deposition to fabricate single-layer graphene-coated smooth titanium substrates and cultured human adipose-derived stem cells as well as bone marrow mesenchymal stem cells on these surfaces, resulting in enhanced cell proliferation, viability, and osteogenic performance [21]. These studies suggest the potential application of a dual-functional modification combining TiO_2_ nanotubes and graphene to elicit synergistic effects and significantly enhance rapid osteogenesis of implants.

Recent research [22] investigated the effects of depositing reduced graphene oxide (rGO) onto TiO_2_ nanotubes via atmospheric plasma treatment, enhancing MC3T3-E1 cell adhesion and proliferation. However, this study did not conduct in-depth exploration related to osteogenesis. There is still a research gap in modifying GO and GQDs on nanotubes and exploring their osteogenic differentiation effects in vivo and in vitro. Cyclic voltammetry (CV) is utilized as a controlled, low-voltage technique that enables sustainable and non-destructive integration of GO/GQDs with the nanotube substrate. Based on the CV method, this study first employs electrodeposition to load GO and GQDs onto the surface of titanium nanotubes, with a focus on eliciting synergistic effects while preserving the structural integrity of the nanotubular architecture. Subsequently, this research aimed to investigate the effects of GO/GQD-deposited TiO_2_ nanotubes on cell proliferation, osteogenic differentiation of rat BMSCs, and early osseointegration through both in vitro and in vivo experiments. The null hypothesis posits that there will be no significant difference between GO/GQD-deposited TiO_2_ nanotubes and untreated controls.

## 2. Materials and Methods

### 2.1. Sample Preparation

According to the previous research method [5], nanotubes (NTs) on the surface of pure titanium were prepared by anodic oxidation. The TiO_2_-NT samples were set as the control group. The electrolyte for the TiO_2_-NT samples was 1 mol/L H_3_PO_4_, 0.5 wt% HF, prepared in deionized water; the electrical parameters were set with a voltage of 15V for 3 h. Based on this, the experimental group used the electrochemical deposition method to deposit graphene oxide (NT-GO group) and graphene oxide quantum dots (NT-GQD group) on the surface of TiO_2_-NT samples.

Before electrodeposition, the formulation of the electrolyte is as follows:

1. Prepare the NT-GO intermediate phase solution: Dissolve 50 mg of graphite oxide dispersion in 100 mL of methanol and ethanol (volume ratio 1:4) under stirring conditions, to obtain an intermediate phase solution with a concentration of 0.5 mg/mL of graphite oxide dispersion.
2. Prepare the NT-GQD intermediate phase solution: Dissolve 10 mg of graphene quantum dots in 100 mL of methanol and ethanol (volume ratio 1:4), to obtain an intermediate phase solution with a concentration of 0.1 mg/mL of graphene quantum dots.
3. Add 20 mg potassium chloride to the above prepared intermediate phase solution. Stir at a constant temperature of 50 °C until it is completely dissolved to obtain an electrolyte containing 0.2 mg/mL of potassium chloride. After adding the conductive salt and additives, the conductivity of the electrolyte can be improved, enabling the electrodeposition process to proceed continuously and rapidly.

The samples were prepared by cyclic voltammetry (CV)(PARSTAT 2273 Advanced Electrochemical System, Princeton Applied Research, Princeton, NJ, USA) using the prepared electrolyte. The voltage window ranged from −1.5 V to +0.1 V. The scanning rate was set as 50 mV/s for 15 min. After the electrodeposition process was completed, the samples were ultrasonically cleaned with absolute ethanol, rinsed with deionized water, and dried by air blowing. The final size of the samples for the cell experiments was 10 mm × 10 mm; the size of the samples for the animal experiments was 2 mm diameter × 6 mm length.

### 2.2. Assessment of Surface Characterization

The surface morphology of the material was examined by scanning electron microscopy (SEM, Sirion 200, FEI Company, Amsterdam, The Netherlands) as well as Raman spectroscopy (LabRAM Solei, HORIBA, Tokyo, Japan) of graphene.

### 2.3. Cell Spreading

In accordance with the ARRIVE guidelines, all experimental protocols involved in this study were approved by the Ethics Committee of the Ninth People’s Hospital Affiliated to Shanghai Jiao Tong University School of Medicine (Approval Number: SH9H-2019-A70-1). BMSCs were isolated from the femurs and tibias of 6-week-old male Sprague Dawley (SD) rats, according to the method described previously [23]. Cells of P2-P4 stages were used for the subsequent experiments. All experiments were independently replicated at least three times. The samples of three groups were sterilized and placed in 24-well plates. Dulbecco’s modified Eagle’s medium (DMEM) with 10% fetal bovine serum was prepared and used for subsequent cell experiments. To characterize the osteogenic differentiation ability of the cells, BMSCs were incubated with osteogenic medium, which comprised high-glucose DMEM supplemented with 10% FBS, 0.01 mM dexamethasone, 10 mM β-glycerophosphate (Sigma-Aldrich, St. Louis, MO, USA), and 0.2 mM L-ascorbic acid (Sigma-Aldrich, St. Louis, MO, USA).

The cells were uniformly inoculated on the surface of the samples at a density of 5*10^3^ cells per well and cultured in an incubator at 37 °C, 5% CO_2_, and 95% relative humidity. After 1 day, the samples were washed with PBS solution carefully and fixed with polyformaldehyde, and the cell morphology was observed using an electron microscope. The spreading of the cell cytoskeleton and cell pseudopodia was recorded.

### 2.4. Assessment of Cell Adhesion and Proliferation

According to the above method, the cells were inoculated on the surface of samples at a density of 5 × 10^4^ cells per well in 24-well plates. On day 1, day 4, and day 7, live/dead cell staining was used to detect the cell activity staining by using the Beyo3D™ Calcein (Calcein Acetoxymethyl Ester)/PI (Propidium Iodide) Cell Viability and Cytotoxicity Assay Kit (Beyotime, Shanghai, China). After removing the culture medium, the cells were washed with PBS. Next, 250 μL of the calcein AM/PI detection solution was added to each well, and the cells were incubated at 37 °C in the dark for 30 min. The staining results of live and dead cells were observed under a fluorescence microscope. Green fluorescence indicated live cells, while red fluorescence indicated dead cells. The MTT assay was employed to analyze the proliferation ability of the cells on the samples. The absorbance (OD) value was measured within the 490 nm wavelength range.

### 2.5. ALP Activity

On day 14, ALP activity staining and Quantitative ALP activity assay were performed to evaluate the osteogenic differentiation of BMSCs by using a BCIP/NBT Alkaline Phosphatase Color Development Kit (Beyotime, Shanghai, China) and an Alkaline Phosphatase Assay Kit (Beyotime, Shanghai, China). The cells were fixed with 4% paraformaldehyde for 30 min. The ALP staining solution was prepared according to the product instructions for ALP staining. The total protein concentration of the cells was semi-quantitatively detected, and the relative ALP activity was calculated.

### 2.6. Formation of Mineralized Nodules

After 21 days of cell culture, the cells were fixed using the same method as described above. Alizarin red staining and semi-quantitative evaluation were used to assess the formation of mineralized nodules in BMSCs by using an Osteoblast Mineralization Nodule Staining Kit (Alizarin Red S Method) (Beyotime, Shanghai, China).

### 2.7. Real-Time PCR

After 7 days and 14 days, cells collected from the surfaces of the five samples were pooled into a single PCR sample to ensure sufficient total RNA for downstream analysis. Total RNA was extracted using a TaKaRa MiniBEST Universal RNA Extraction kit (Takara Bio Inc., Tokyo, Japan) according to the manufacturer’s instructions, and cDNA was synthesized with a TaKaRa PrimeScript 1st Strand cDNA Synthesis kit (Takara Bio Inc., Tokyo, Japan). The expressions of osteogenesis-related genes *ALP*, *OSX*, *VEGF*, and *OCN* were analyzed using a real-time PCR system (Bio-Rad, Hercules, CA, USA). The relative expression level was normalized to that of the internal control gene β-actin. The expressions of each group of genes were analyzed using the 2-ΔΔCt method. The primer sequences are listed in Appendix A Table A1.

### 2.8. Animal Experiments

Nine male 6-week-old SD rats (weight: ~200–250 g) were provided by the Animal Laboratory Center of Shanghai Ninth People’s Hospital Affiliated with Shanghai Jiao Tong University. Animals were housed in standard polycarbonate cages (*n* = 2–3 per cage) in a controlled, specific pathogen-free (SPF) environment and under standard conditions (12 h light/dark cycle, 22 °C, 50% humidity) with ad libitum access to food and water.

All surgical procedures and sample analyses were conducted in a blinded and consistent manner to minimize assessment bias.

The cylindrical implants of each group with a diameter of 2 mm and a length of 6 mm were sterilized for the animal experiments. Next, 2% pentobarbital sodium solution was injected into the abdominal cavity of SPF six-week-old male SD rats. At the position of the femur and tibia of the lower limbs of the rats, the epiphyseal ends of the femur and tibia were fully exposed. The implantation holes (∅ = 2.0 mm) were prepared in the distal femur along the direction of the medullary cavity. For in vivo studies, nine animals were randomly assigned to 3 experimental groups using a computer-generated random number sequence. The groups were coded as X, Y, and Z. The rats were marked with sterile ear tags bearing unique codes following a “group-individual” method (i.e., X-1; Y-2; Z-3). To minimize assessment bias, all subsequent procedures, including the surgery procedure and sample collection, were performed by investigators blinded to the group codes. Each rat received one implant randomly in a single femoral epiphysis. The wound was carefully closed with sutures. The final data analysis only included animals that successfully completed the entire experimental protocol. Any animals showing severe, unalleviated pain or complications (e.g., infection unresponsive to treatment, fracture) that meet pre-defined humane endpoints were euthanized immediately and excluded from the final analysis.

Sequence fluorescence labeling was applied to observe the formation of new bone at different stages. Fluorescent markers, including tetracycline (TE), alizarin red (AL), and calcein (CA), can bind to calcium ions in bone tissue and form complexes that deposit in situ. Through sequential fluorescent labeling, the rate of calcium ion deposition around the implant can be compared by calculating the fluorescent areas at different time points, enabling an assessment of new bone formation on the material surface. Subsequently, 2 weeks after the operation, tetracycline solution (TE) was injected intraperitoneally into the experimental rats at a dose of 20 mg/kg body weight to mark them with yellow fluorescence; 4 weeks after the operation, alizarin red solution (AL) was injected intraperitoneally at a dose of 30 mg/kg body weight to mark them with red fluorescence; 6 weeks after the operation, calcein solution (CA) was injected intraperitoneally at a dose of 25 mg/kg body weight to mark them with green fluorescence; 8 weeks after the operation, rats were sacrificed by intraperitoneal injection of excessive pentobarbital sodium, and the bilateral femurs were separated. The bone specimens containing the implants were carefully removed and fixed in 4% paraformaldehyde solution.

### 2.9. Micro-CT Scanning

Micro-CT (Scano Medical AG, Wangen-Brüttisellen, Switzerland) was used to scan the bone specimens containing implants, calculating the new bone volume (BV) within 0.25 mm around the implant, the bone volume fraction (BV/TV), and the number of trabeculae (Tb.N).

### 2.10. Hard-Tissue Sections

After the samples underwent micro-CT scanning were fixed, they were sequentially placed in 70%, 75%, 80%, 90%, 95%, 100%, and 100% ethanol solutions for 24 h each for gradient dehydration; they were then treated with pure xylene for 8 h for transparency and embedded, and the specimens were cut longitudinally along the implant axis using a Leica hard-tissue sectioning machine (Leica Microsystems, Wetzlar, Germany) to complete the section preparation. The fluorescence-labeled new bone formation around the implant was observed under a laser confocal microscope, and the areas of each color were calculated using ImageJ (https://imagej.net/ij/, National Institutes of Health, Bethesda, MD, USA) image analysis software.

### 2.11. Statistical Analysis

The results were represented with a mean value ± standard deviation from at least three independent repetitions. One-way ANOVA and Tukey’s post hoc test were used to evaluate the statistical significance level. Normality and homogeneity of variance were tested using Shapiro–Wilk and Levene’s tests. Non-parametric tests were used if assumptions were violated. Furthermore, *p* < 0.05 indicates a significant difference.

## 3. Results

### 3.1. Surface Characterization

**Figure 1:**
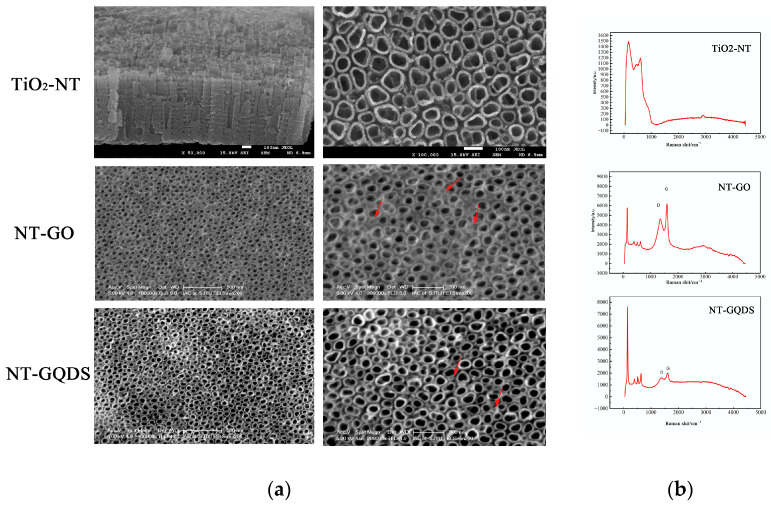
Surface characterization. (**a**) SEM results of each group (the arrow indicates the electrodeposited GO and GQDs); (**b**) Raman spectrum.

**Figure A1:**
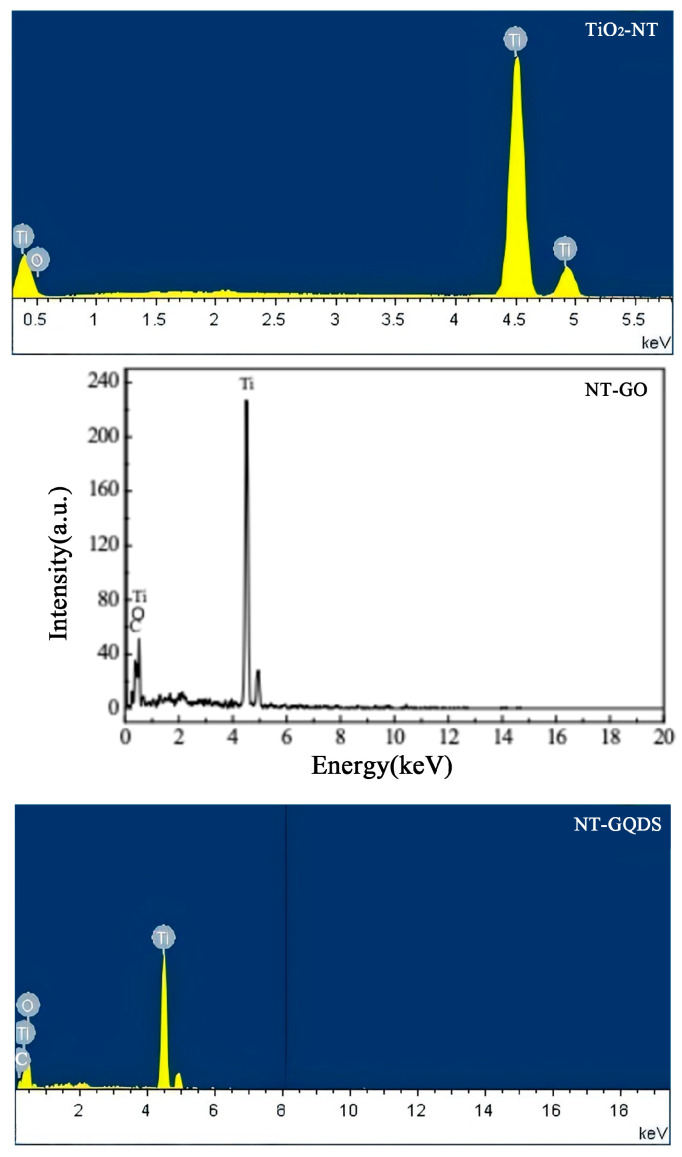
EDS analysis of each sample.

The SEM results show that the anodic oxidation method can successfully prepare nanotubes on the titanium surface, which is consistent with our previous research [24]. Meanwhile, graphene oxide and quantum dots were, respectively, dispersed on the surface of the nanotubes, as indicated by the arrows in Figure 1a. Information such as the structural defects of graphene (D peak) and the in-plane vibration of sp^2^ carbon atoms (G peak) is well reflected in the Raman spectrum (Figure 1b). After electrodepositing GO and GQDs using the CV method, a clear double-peak structure appeared on the surface of the modified samples. The typical D peak (1200–1450 cm^−1^) and G peak (1500–1700 cm^−1^) indicate that adding the graphene intermediate phase can enable electrodeposition through the CV method at low temperature and low pressure. The corresponding values of D peak and G peak on the Y axis in the NT-GO group were higher than those in the NT-GQD group, indicating a higher GO concentration on the NT-GO surface than on the NT-GQD surface. In contrast, these two peaks were not observed on the TiO_2_-NTs. These results indicate that we successfully coated graphene onto the TiO_2_-NT surface. EDS analysis of each group (Figure A1) confirmed the change in carbon composition before and after electrodeposition.

### 3.2. Cell Adhesion and Proliferation

**Figure 2:**
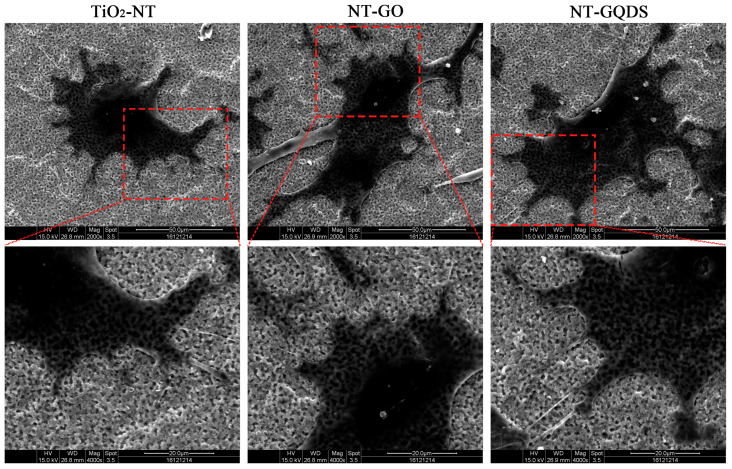
SEM result of cell adhesion and spreading.

Each group of cells exhibited the characteristic of having numerous pseudopodia extending on the material surface, and they were able to firmly adhere to the material surface. From the SEM in Figure 2, we can observe that the cells in the NT-GO group and the NT-GQD group had a larger spreading area and more-dense pseudopodia than the TiO_2_-NT group.

**Figure 3:**
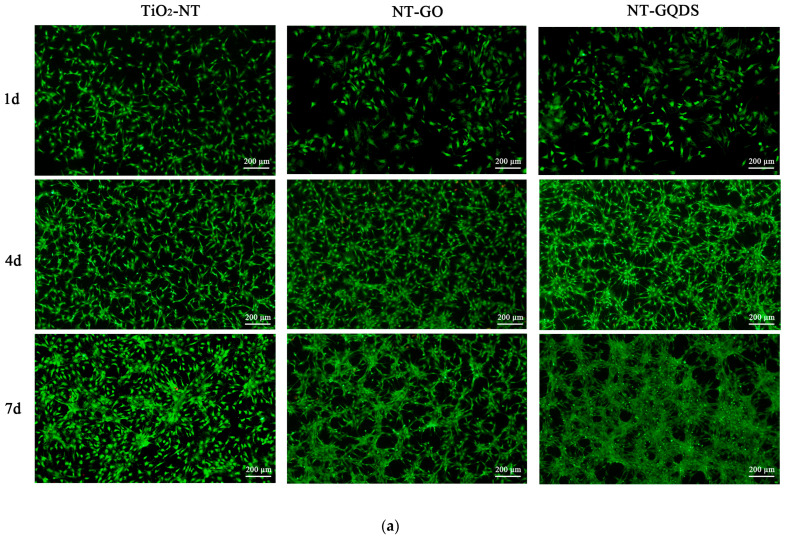

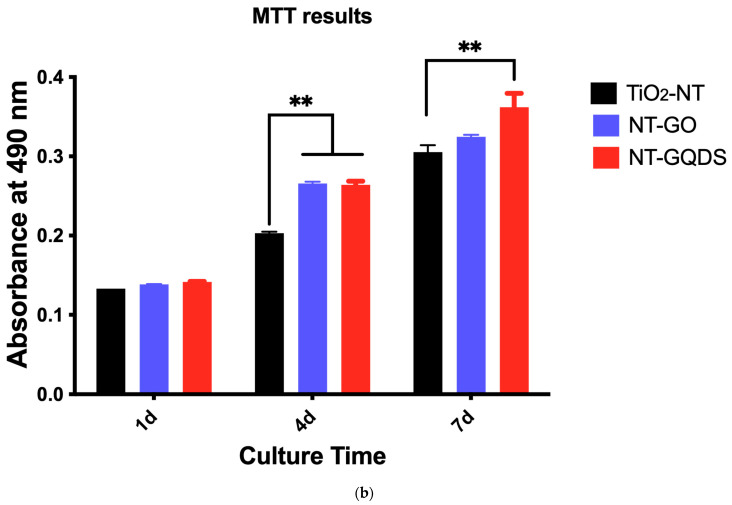
Cell viability staining and MTT testing. (**a**) Live/dead cell staining. Green fluorescence indicated live cells, while red fluorescence indicated dead cells; (**b**) MTT results (** for *p* < 0.01) (*n* = 3).

Live/dead cell staining (Figure 3a) showed that the surfaces of each group of materials did not have any toxic effect on the BMSCs. The MTT results (Figure 3b) indicated that on day 1 of culture, there was no difference in cell viability among the three groups. On day 4 of culture, the cell viability of the NT-GO and NT-GQD groups was significantly higher than that of the TiO_2_-NT group. On day 7 of culture, there was no difference in cell viability between the NT-GO group and the control group, but the cell viability in the NT-GQD group was still significantly higher than that in the control group (*p* < 0.01).

### 3.3. Osteogenic Differentiation

**Figure 4:**
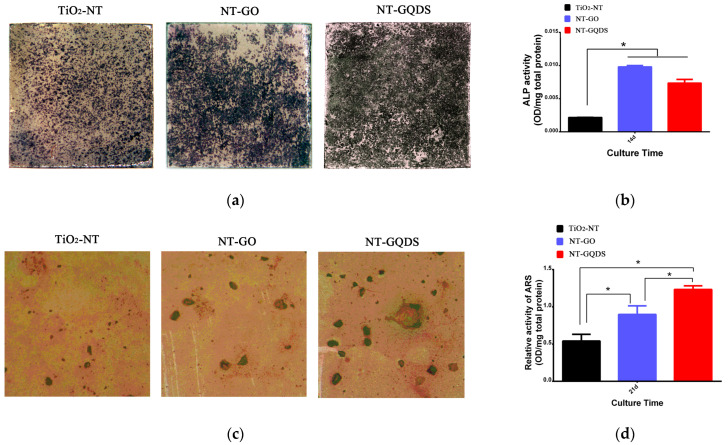
ALP activity and formation of mineralized nodules: (**a**) ALP staining; (**b**) Quantitative ALP activity assay; (**c**) alizarin red staining; (**d**) Quantitative ARS assay. * for *p* < 0.05 (*n* = 3).

As shown in Figure 4a, the ALP staining of all groups showed intense ALP staining on day 7 of culture. The Quantitative ALP activity assay (Figure 4b) indicated that the ALP activity of the NT-GO group and the NT-GQD group was significantly higher than that of the control group. This suggests that both the electrodeposition of GO and GQDs on the surface of the nanotubes can enhance the osteogenic differentiation performance of BMSCs.

According to the alizarin red staining (Figure 4c), there were obvious differences in the staining of mineralized nodules. The NT-GQD group showed larger mineralized nodules than the TiO_2_-NT group. The semi-quantitative results of mineralized nodules (Figure 4d) indicated NT-GQD group > NT-GO group > TiO_2_ control group.

**Figure 5:**
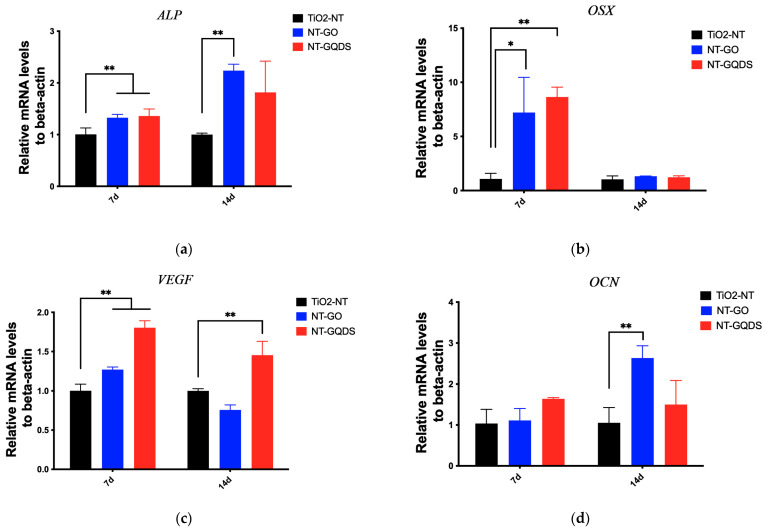
PCR results. Osteogenic-related genes of (**a**) *ALP*; (**b**) *OSX*; (**c**) *VEGF*; (**d**) *OCN* (* *p* < 0.05; ** *p* < 0.01) (*n* = 3).

The PCR results are shown in Figure 5. On the 7th day of culture, the expressions of *ALP*, *OSX*, and *VEGF* in the NT-GO group and NT-GQD group were significantly higher than those in the control group, while *OCN* showed no difference in the NT-GO and NT-GQD compared with the TiO_2_-NT group. On the 14th day of culture, the expression of *ALP* in the NT-GO group was higher than that in the control group; the expression of *OSX* shown no difference compare with that in the control group; the expression of *VEGF* in NT-GQD was significantly upregulated compared with the control group, and the expression of *OCN* in the NT-GO group was significantly upregulated compared with that in the control group.

### 3.4. In Vivo Osseointegration of Implant

**Figure 6:**
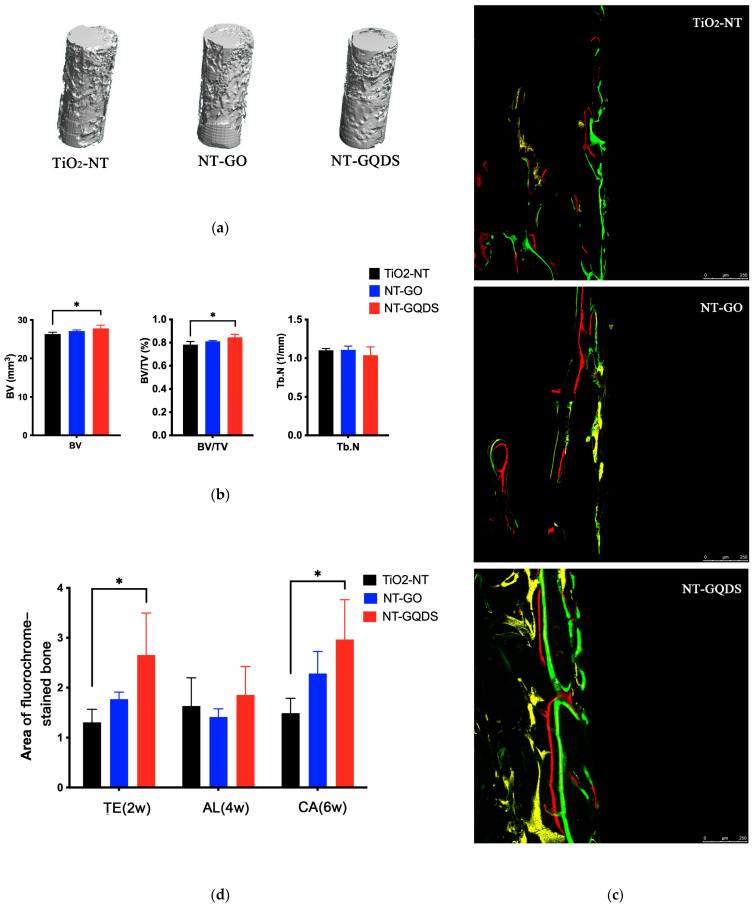
Micro-CT testing and sequence fluorescence labeling. (**a**) Implant and surrounding bone reconstruction (0.25 mm around the implant) of 8 weeks; (**b**) analysis of BV, BV/TV, and Tb.N; (**c**) sequence fluorescence labeling, TE (yellow, 2 weeks), AL (red, 4 weeks), CA (green, 6 weeks); (**d**) analysis of area of fluorochrome-stained bone. * *p* < 0.05; *n* = 3.

The results of the in vivo experiments are presented in Figure 6. After analyzing the micro-CT results of each group of samples, the statistical results indicated that both the BV and BV/TV of NT-GQDs significantly increased, suggesting that NT-GQDs can promote new bone formation around the implants in rats. The sequential fluorescence results showed that the fluorescence area of TE(2W) was significantly greater than that of the control group, with a statistical difference. Moreover, the yellow fluorescence area of the NT-GO group was also higher than that of the control group, indicating that both NT-GO and NT-GQDs can promote new bone formation in the early postoperative stage, with the latter showing a more significant effect. The results of the statistical fluorescence area (Figure 6d) also confirm this point. Additionally, the fluorescence area of CA(6W) was also higher than that of the control group. This indicates that the NT-GQD implant can continuously promote rapid osseointegration around the implant. Results of a post hoc power analysis based on the obtained in vivo data regarding the results with statistical significance are shown in Appendix A Table A2.

## 4. Discussion

Orthopedic and dental implants necessitate titanium surfaces with multifunctional properties, particularly in cases in which bone regeneration is compromised due to aging or pathological conditions [3]. The success rate of titanium implant surgery exceeds 90%; however, approximately 10% of implants still fail [25,26]. Evidence from studies indicates that 10–20% of such failures are attributable to bacterial infection and inadequate osseointegration during the implantation process [27]. The surface characteristics of titanium implants play a critical role in determining osseointegration efficacy [28]. It is meaningful that our research demonstrates that electrodepositing GO and GQDs onto the surface of nanotubes promotes the adhesion, proliferation, and osteogenic differentiation of BMSCs. Furthermore, NT-GQDs enhance both the BV and the BV/TV ratio of newly formed bone surrounding the implant. Our previous studies showed that the TiO_2_ nanotube structure is uniform, similar to natural cancellous bone, and exhibits strong osteoinductive, osteoconductive, and biomimetic properties [5]. The dual-functional modification combining TiO_2_ nanotubes and GO/GQDs has been confirmed to exert synergistic effects, significantly enhancing the rapid osteogenesis around implants. Therefore, the null hypotheses were rejected.

By employing the environmentally friendly CV method, dispersed GO and GQDs were successfully electrodeposited onto nanotubes under a low applied voltage (−1.5 V to +0.1 V), avoiding ion dissolution and preventing nanotube damage. Furthermore, the addition of potassium chloride (KCl) to the electrolyte improves conductivity, enabled continuous and rapid deposition at low voltage while offering advantages of low energy consumption, high safety, and ease of operation. Raman spectroscopy showed more GO than GQDs on the NT surface. This is likely because Raman spectroscopy detects cumulative lattice vibration signals of carbon materials, such as the D and G peaks. GO possesses a two-dimensional sheet-like structure that can form a relatively continuous, dense, and thick film on titanium nanotubes via π–π stacking or electrostatic interactions [10]. Due to its high carbon atom density, GO exhibits a strong Raman signal. In contrast, GQDs are zero-dimensional carbon nanomaterials with a lateral size less than 20 nm [29]. Even under electrochemical deposition, GQDs tend to attach to the surface and interior of nanotubes in isolated or discontinuous configurations, resulting in a much lower density of effective carbon atoms per unit area compared to GO. During CV scanning, GO—rich in oxygen-containing functional groups (e.g., carboxyl and epoxy groups)—undergoes electrochemical reduction at negative potentials, leading to direct deposition on the electrode. In contrast, GQDs may adsorb at negative potentials but could desorb back into the solution when potentials shift to positive or open-circuit conditions, resulting in a lower deposition efficiency than that for GO.

NT-GO and NT-GQDs fabricated via the CV method exhibit superior biocompatibility without evident cytotoxicity. Although the cytotoxicity of GO is known to be dose dependent, studies have shown that GO and its derivatives can promote BMSC proliferation and differentiation at concentrations ranging from 0 to 50 μg/mL or higher [14,30,31]. At concentrations below 1 μg/mL, GQDs significantly enhance BMSC viability and proliferation, with 0.1 μg/mL exhibiting the most pronounced effect on osteogenic differentiation [14]. These findings highlight the concentration-dependent biological effects of GO-based materials in aqueous solutions. In contrast, the nanotube-based composites prepared in this study display consistent biocompatibility, likely due to the stable immobilization of GO and GQDs on the nanotube surface. Due to its inherent inert carbon-based properties, graphene exhibits high chemical and physical stability and is non-toxic [32]. It has been similarly reported that graphene-coated substrates are non-toxic to MSCs and human osteoblasts [33]. Due to the above-mentioned electrochemical deposition characteristics, NT-GO is more inclined to affect cells through physical and chemical properties, while NT-GQDs tend to influence cells through the nano-size effect. The highest proliferation of BMSC was observed for the NT-GQDs. The synergistic effect of nano-scale topological structures of NT-GQDs, basically retaining the original nanotube array structure, alters the local mechanical properties of the material surface, provides more anchor sites for the pseudopodia of cells, and creates a microenvironment more conducive to cell spreading and adhesion (Figure 2). Cell spreading is a prerequisite for cells to enter the proliferation cycle. This topological structure effect might be the reason for the difference between NT-GO and NT-GQDs.

Besides proliferation, cell adhesion assays yielded superior results. SEM of cell spreading in this study revealed that cells exhibited an extended morphology, as shown in Figure 2. The presence of GO and GQDs facilitated cell spreading over a larger surface area. Furthermore, 1 day after cell inoculation, BMSCs displayed more-extensive and densely developed pseudopodia than the TiO_2_-NT group. Previous studies have reported enhanced expression of adhesion plaques at the tips of cellular pseudopodia in graphene-based materials. Focal adhesions (FAs), which are structural proteins associated with adhesion plaques [21], may be promoted by the high surface energy of graphene, providing a more favorable substrate environment for human mesenchymal stem cells (hMSCs). GDs possess a large specific surface area and abundant oxygen-containing functional groups, enhancing protein adsorption, enabling electrostatic binding, and promoting strong cell membrane interactions and widespread cellular adhesion [10]. Furthermore, GO features a two-dimensional honeycomb lattice composed of carbon atoms, while GQDs are zero-dimensional carbon nanomaterials with lateral dimensions below 20 nm [29]. These structural characteristics may enhance the material’s hydrophilicity, thereby promoting BMSC adhesion on the surface.

Graphene maintains the multi-differentiating potentiality [34] and also promotes the osteogenic differentiation potential [20,35]. Implant osseointegration relies on the promotion of cell proliferation, adhesion, angiogenesis, and osteogenic differentiation [36]. In our study, early osteogenic markers *ALP* and *OSX* were upregulated in the NT-GO group and NT-GQD group compared with the TiO_2_-NT group. In addition, the angiogenic marker *VEGF* and the late-stage osteogenic marker *OCN* were upregulated in the NT-GQD group. This suggests that the NT-GQD group may offer greater advantages in long-term in vivo studies using large animal models. Elevated *OCN* expression is associated with matrix mineralization and promotes mineral deposition, which has been confirmed in our ARS assay and in vivo animal experiments.

To explain the role of GO/GQDs in osteogenesis, two mechanisms have been proposed. First, increased substrate stiffness, modified by depositing GO/GQDs, may promote osteogenesis by influencing cellular mechanotransduction [37]. It has been reported that substrate stiffness is a potent regulator of pre-osteoblasts and fibroblasts, capable of promoting osteogenic differentiation in pre-osteoblasts [38]. The resulting mechanical tension activates downstream effectors such as YAP/TAZ, which are known to translocate into the nucleus and collaborate with osteogenic-related genes to drive osteogenic commitment. Second, enhanced electrical conductivity of the substrate can facilitate calcium ion influx, thereby supporting bone differentiation and biomineralization [37,39]. The use of GQDs has been shown to increase mineralized nodule formation, stimulate biomineralization, and accelerate bone regeneration [40], findings consistent with our alizarin red S (ARS) staining results. GQDs promote the osteogenic differentiation of mesenchymal stem cells (MSCs) at multiple levels, including gene activation, mRNA expression, and protein synthesis [21]. In vivo results demonstrate that NTs and GQDs are effectively integrated in implants, exhibiting synergistic effects that surpass those of NTs alone. Regarding the mechanism by which the graphene oxide family promotes osteogenesis, Xu et al. [14] reported that GQDs improved the osteogenic differentiation ability of BMSCs by activating the Wnt/β-catenin signaling pathway. Alexander Halim et al. [41] showed that the osteoinductive and antioxidative effects of low-dose GO nanosheets were achieved through the activation of JNK and FoxO1 signaling pathways. Research on the modification of metal surfaces, particularly titanium, remains limited. Table 1 summarizes the biological effects and underlying mechanisms of graphene and its derivatives in titanium surface modification. A similar approach [22] combining graphene oxide with nanotubes has been investigated primarily for pre-osteoblastic cell proliferation but lacks in-depth exploration of osteogenic differentiation and osseointegration. The specific molecular mechanism of the synergistic TiO_2_-NT/GQD system remains to be further studied.

While this study demonstrates the promising potential of the NT-GQD modification for enhancing early osteogenesis, several limitations should be acknowledged. The study focused on early bone healing (8 weeks), so long-term coating stability, bio-durability, and bone–implant integration strength remain to be evaluated. The rat model and small sample size (*n* = 3 per group) limit generalizability, as bone healing in larger animals or humans may differ. However, a post hoc power analysis based on the obtained in vivo data regarding the results with statistical significance was performed (Table A2). A target statistical power of at least 80% ensures the reliability of the study results. The power of BV/TV and area of TE reached 86.51% and 80.16%, respectively. The current experimental design is adequate, and the findings remain reliable and biologically meaningful. Future work will include long-term implantation in large-animal models and biomechanical pull-out tests to evaluate bone–implant integration. In addition, we did not conduct a quantitative release study to assess GO and GQD stability on the NT surface, and our tests used only a limited range of GO/GQD concentrations. Different concentrations may produce better biological outcomes. Both nanotubes and graphene serve as highly effective sustained-release carriers. GDs function not only as substrates for metal nanoparticles but also as reservoirs for dissolved metal ions, thereby preventing nanoparticle aggregation, reducing metallic toxicity, and enabling the controlled release of metal ions [10,45]. These properties offer promising potential for future applications in controlled drug delivery systems, including antibacterial agents and therapeutic delivery platforms, which may help prevent bacterial infections and implant failure. Further research is warranted to fully understand and harness any additional beneficial effects.

## 5. Conclusions

Our study establishes the synergistic NT/GQD system as a promising strategy for advancing the design of orthopedic and dental implants. This modification not only enhances the biocompatibility of implant surfaces but also actively promotes early osteogenesis through the synergistic enhancement of BMSC adhesion and osteogenic differentiation. These findings highlight the substantial translational potential of NT-GQD in accelerating osseointegration, thereby improving implant stability and long-term clinical success. Future studies will focus on validating these results in large-animal models to bridge the gap between fundamental research and clinical application.

## Figures and Tables

**Table 1:** Biological effects and mechanisms of graphene and its derivatives on the surface modification of titanium.

Graphene	Substrate	Coating Method	Outcomes	Mechanism
GO	(SLA)-treated Ti	Ultrasonic atomization spraying technique	Promoted cell proliferation, adhesion, diffusion, and osteogenic differentiation	FAK/P38 signaling pathways [42]
rGO	Ti	Meniscus-dragging deposition	Promoted cell proliferation, ALP activity, and matrix mineralization	Decrease in the surface roughness and contact angle [43]
rGO	(SLA)-treated Ti	Peptide bonds	Promoted osteogenesis	Absorption of exogenous proteins [44]
Graphene films	Ti6Al4V	Physical adsorption	Promoted the adhesion and early extension of BMSCs	Surface morphology [20]
rGO	TiO_2_ nanotube	Atmospheric plasma deposition	Increased the initial adhesion and proliferation of pre-osteoblastic cells	Increase in contact angle [22]
GQDS	(SLA)-treated Ti6Al4V	Immersion	Accelerated new bone formation	Activation of the Wnt/β-catenin signaling pathway [14]

GO; graphene oxide; Ti: Titanium; rGO: reduced GO; SLA: sandblasted, large grit, and acid etched; TiO_2_: Titanium dioxide; GQDS: graphene quantum dots.

## Data Availability

The original contributions presented in the study are included in the article; further inquiries can be directed to the corresponding authors.

## Abbreviations

The following abbreviations are used in this manuscript:

BMSCs	Bone marrow-derived mesenchymal stem cells
NT	Nanotubes
GO	Graphene oxide
GQDs	Graphene quantum dots
GDs	Graphene and its derivatives
SEM	Scanning electron microscope
CV	Cyclic voltammetry
TiO_2_	Titanium dioxide

## Appendix A

**Table A1:** The primer sequences of the related genes in the study.

Genes	Primer Sequence
*β-actin*	F: CACCCGCGAGTACAACCTTC
	R: CCCATACCCACCATCACACC
*ALP*	F: CACGTTGACTGTGGTTACTGCTGA
	R: CCTTGTAACCAGGCCCGTTG
*Osterix*	F: CTATGCCAATGACTACCCACCC
	R: CTGCCCACCACCTAACCAA
*VEGF*	F: TTGAGTTGGGAGGAGGATGT
	R: TGGCAGGCAAACAGACTTC
*OCN*	F: GCCCTGACTGCATTCTGCCTCT
	R: TCACCACCTTACTGCCCTCCTG

**Table A2:** Post hoc power analysis based on the obtained in vivo data regarding the results with statistical significance.

Items	Effect Size (Cohen’s f)	Power (1-β Err Prob)
BV	1.28	75.13%
BV/TV	1.482	86.51%
Area of TE	1.36	80.16%
Area of CA	1.31	77.09%
